# Extraordinarily potent proinflammatory properties of lactoferrin-containing immunocomplexes against human monocytes and macrophages

**DOI:** 10.1038/s41598-017-04275-7

**Published:** 2017-06-26

**Authors:** Lulu Hu, Xiaomin Hu, Kai Long, Chenhui Gao, Hong-Liang Dong, Qiao Zhong, Xiao-Ming Gao, Fang-Yuan Gong

**Affiliations:** 1 Institute of Biology and Medical Sciences, School of Biology and Basic Medical Sciences, Soochow University, Suzhou, China; 2 Department of Immunology, Peking University Health Science Center, Beijing, China; 3 Department of Physiology, Jiujiang College, Jiangxi Province, China

## Abstract

Lactoferrin (LTF), an important first line defense molecule against infection, is a common target for humoral autoimmune reactions in humans. Since LTF is a multifunctional protein capable of activating innate immune cells via various surface receptors, we hypothesized that LTF-containing immune complexes (ICs) (LTF-ICs), likely formed in patients with high titer anti-LTF autoantibodies, could possess unique monocyte/macrophage-activating properties compared with other ICs. ELISA analysis on serum samples from rheumatoid arthritis (RA) patients (n = 80) and healthy controls (n = 35) for anti-LTF autoantibodies confirmed a positive correlation between circulating LTF-specific IgG and RA. ICs between human LTF and LTF-specific IgG purified from patient sera or immunized rabbits and mice, but not control ICs, LTF or Abs alone, elicited strong production of TNF-α and IL-1β by freshly fractionated human peripheral blood monocytes and monocytes-derived macrophages. Furthermore, LTF-ICs utilized both membrane-anchored CD14 and CD32a (FcγRIIa) to trigger monocyte activation in an internalization-, Toll-like receptor (TLR)4- and TLR9-dependent manner, and also that LTF-IC-induced cytokine production was blocked by specific inhibitors of caspase-1, NF-κB and MAPK. These results uncover a possible pathway for LTF-ICs perpetuating local inflammation and contributing to the pathogenesis of autoimmune diseases by triggering activation of infiltrating monocytes or tissue macrophages *in vivo*.

## Introduction

Lactoferrin (LTF) is an ~80 kDa multifunctional iron-binding glycoprotein of the transferrin family found in most mammalian exocrine secretions as well as secondary granules of neutrophils^1^. Besides its physiological roles in the process of iron homeostasis, LTF also functions as an important first line defense molecule against microbial invasion^1–3^. Its ability to sequester bacterial lipopolysaccharides (LPS), a prototypic pathogen-associated molecular pattern (PAMP), and modulate endotoxin shock *in vivo* has been extensively studied^2–6^. LTF can directly interact with dendritic cells (DC) and monocytes/macrophages that are of critical importance for the maintenance of tissue homeostasis and defense against microbial infection, modulating their functional roles in inflammatory and infectious processes^7–12^.

Human LTF (huLTF) is known to be a target of dysregulated humoral autoimmune attack *in vivo*, although detailed molecular mechanisms for the generation of anti-LTF autoantibodies in patients are unclear. LTF-specific autoantibodies (LTF-Abs) are found in sera of patients with autoimmune disorders such as systemic lupus erythematosus (SLE), rheumatoid arthritis (RA), ulcerative colitis, Crohn’s disease or anti-neutrophil cytoplasmic Ab (ANCA)-positive autoimmune vasculitis^13–19^. Although concentration of LTF in circulation is normally below 1 μg/ml, it can be significantly raised following tissue inflammation or injury. In the case of inflamed rheumatoid synovial fluid of RA patients, for instance, LTF concentration can reach 70 µg/ml^20, 21^. Thus, LTF-containing immunocomplexes (ICs) can be the direct and real-time products of anti-LTF humoral responses in humans, which could potentially be pathogenic unless efficiently removed by phagocytosis. The Fc portion of immunoglobulin (Ig) heavy chains in ICs can directly interact with innate immune cells expressing various Fc receptors (FcRs). Results from experiments employing transgenic or gene knockout mice suggest that IC triggering of the FcγRs on macrophages, which leads to secretion of leukotriens, prostaglandin E2 (PGE2) and proinflammatory cytokines such as TNF-α and IL-1β, plays a more important role than complement activation in the pathogenesis of autoimmune arthritis^22, 23^.

ICs exhibit considerably diverse (even opposite) immunological functions both *in vivo* and *in vitro*. For example, chicken ovalbumin (OVA)-containing ICs modulated human monocyte expression of molecules (CD14, MHC II, ICAM-1) involved in inflammation and immune response^24^ and inhibited LPS response of murine macrophages via FcγRIIb and production of PGE2^25, 26^. Janczy *et al*. recently reported that ligation of activating FcγRs by ICs suppressed IL-1 secretion by antigen presenting cells (APCs) through inhibition of inflammasome activation^27^. On the other hand, ICs containing dsDNA and citrilinated fibrinogen are potent activators of DCs and monocytes/macrophages and may play pivotal roles in initiating overt inflammatory tissue damage in disease conditions^28–31^. Identification and characterization of such biologically active ICs in patients will help to understand the pathogenesis of systemic autoimmune disorders. In the present study, we set out to investigate the pro-inflammatory potentials of LTF-ICs against human monocytes/macrophages.

## Results

### Anti-LTF Abs in RA sera

**Figure 1:**
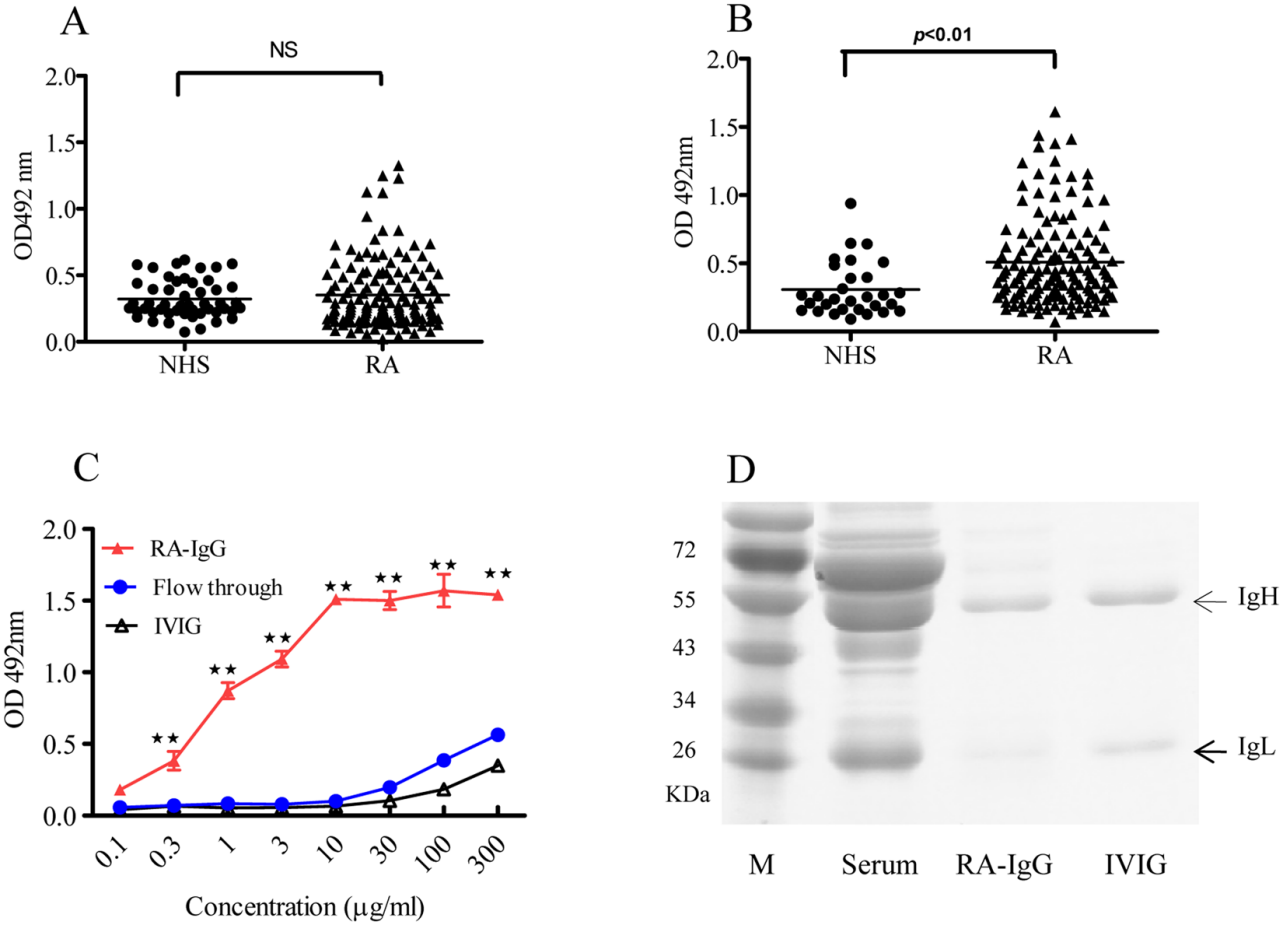
Anti-LTF Abs in RA sera. Serum samples from healthy adults (NHS, n = 35) and patients with RA (n = 80) were individually 1/200 diluted and assayed in huLTF-based ELISAs for detection of huLTF-specific IgM (**A**) and IgG (**B**) Abs. The detection Abs were HRP-conjugated goat-anti-human IgM or IgG with OPD as substrate. Values are the mean OD492 nm ± SEM from triplicate wells. Horizontal lines represent group averages. *NS*: not statistically significant. RA sera with highest levels of anti-LTF IgG were selected and pooled for affinity purification of human anti-LTF IgG autoantibodies using Protein A- and huLTF-Sepharose 4B columns. The resultant huLTF-specific IgG (RA-IgG) was compared with the sample of flow through and also IVIG (commercial) in huLTF-based ELISAs, **p < 0.01 compared with IVIG control (**C**). Samples of RA sera, RA-IgG and IVIG were also analyzed by Coomassie blue-stained SDS-PAGE 10% gel (**D**).

Serum samples from 80 RA patients and 35 healthy subjects (NHS) were analyzed for levels of IgM and IgG against huLTF using ELISAs (Fig. 1A and B). The data acquired has a normal distribution, and for anti-LTF IgG, but not IgM, the difference between RA and NHS groups was statistically significant (*p* < 0.01). When cutoff value was set at 0.43 (OD492 nm), 75% and 20% of the sera were positive for LTF-specific IgG in the RA and NHS groups, respectively. RA sera with highest levels of anti-LTF IgG were selected for affinity purification of human anti-LTF autoantibodies, and the resultant products (RA-IgG, mostly IgG1), characterized using huLTF-based ELISA (Fig. 1C) and SDS-PAGE electrophoresis (Fig. 1D), were subjected to subsequent functional analysis (see below).

### LTF-ICs, but not huLTF or LTF-Abs alone or control ICs, effectively induce TNF-α and IL-1β production by human monocytes *in vitro*

**Figure 2:**
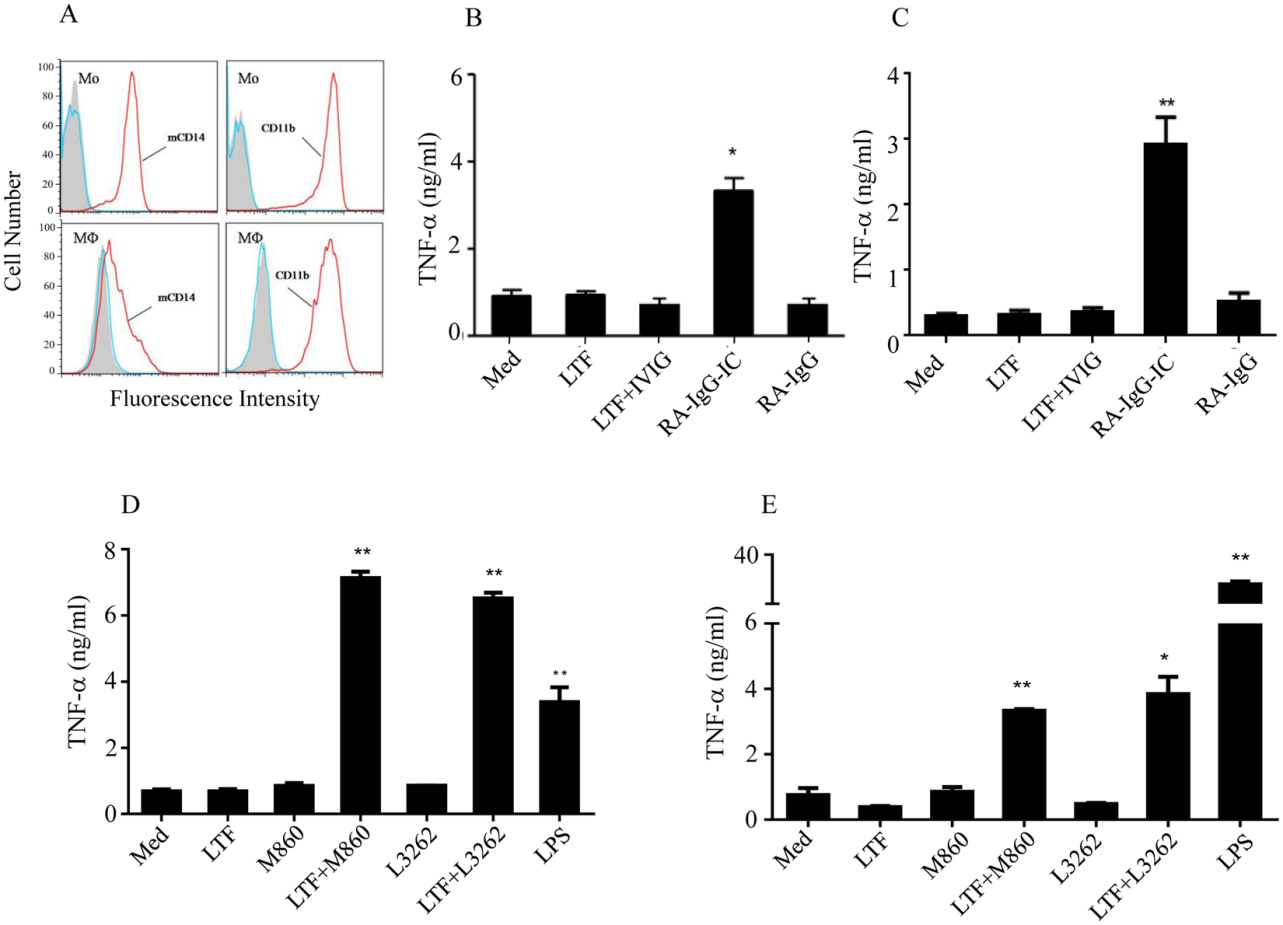
Synergistic effect between huLTF and LTF-Abs. (**A**) Purified human monocytes (Mo, upper panels) and differentiated macrophages (M_Ф_, lower panels) were stained with FITC-conjugated mAbs against human CD14 or CD11b followed by FACS analysis. (**B**) Freshly fractionated human monocytes and (**C**) human monocytes-differentiated macrophages were incubated with 15 μg/ml huLTF, or RA-IgG, or RA-IgG-IC (mixture of huLTF plus RA-IgG), or huLTF plus IVIG (15 μg/ml for each protein), for 24 h. (**D**) Fractionated human monocytes and (**E**) human monocytes-differentiated macrophages were incubated with 15 μg/ml huLTF, or mouse anti-huLTF mAb M860, or rabbit anti-huLTF polyclonal IgG L3262, or a mixture of huLTF plus M860 (*LTF* + *M860*) or L3262 (*LTF* + *L3262*) for 24 h. Cells cultured in medium only (*Med*) or stimulated with 3 μg/ml LPS were included as controls. TNF-α concentration in the culture supernatant was then determined by ELISA. Data are mean concentration ± SEM from triplicate wells and these are representatives of at least 3 independent experiments. *p < 0.05, **p < 0.01 compared with medium controls.

Monocytes in the peripheral blood differentiate into macrophages following their homing to tissues; and upon activation produce cytokines such as TNF-α and IL-1β that mediate much of the inflammatory pathology *in vivo*. The reported effectiveness of soluble huLTF to activate human monocytes/macrophages is controversial^7–11^. In this study, human peripheral blood CD14^+^CD11b^+^ monocytes (Fig. 2A, upper panels), freshly fractionated from PBMCs of healthy donors, were stimulated with huLTF in the presence or absence of RA-IgG for 24 h, followed by ELISA quantitation of TNF-α in the culture supernatant. Treatment of human monocytes with 1:1 mixture of huLTF and RA-IgG (RA-IgG-IC) resulted in significant TNF-α secretion compared to that treated with huLTF, RA-IgG or a mixture of huLTF with normal IgG (IVIG), which is similar to that cultured in medium alone (Fig. 2B). These results indicate that IC formation between huLTF and specific IgG is essential for the activation of monocytes to produce proinflammatory cytokines. Moreover, huLTF-specific IgG from rabbits (L3262, polyclonal IgG) and mice (M860, mAb of IgG1 subclass)^32^ were as effective as, if not better than, RA-IgG in synergizing huLTF in the activation of human monocytes (Fig. 2D). Given that both monocytes and macrophages accumulate in inflamed joints of RA patients, we next addressed the question whether macrophages are similarly responsive as monocytes to LTF-IC stimulation. Human macrophages differentiated from monocytes *in vitro* by cytokine stimulation for 7 days (Fig. 2A, lower panels) were subjected to LTF-IC stimulation. Like monocytes, these macrophages responded to RA-IgG-IC, LTF + L3262 and LTF + M860, but not to LTF alone or in combination with control Abs (Fig. 2C and E).

**Figure 3:**
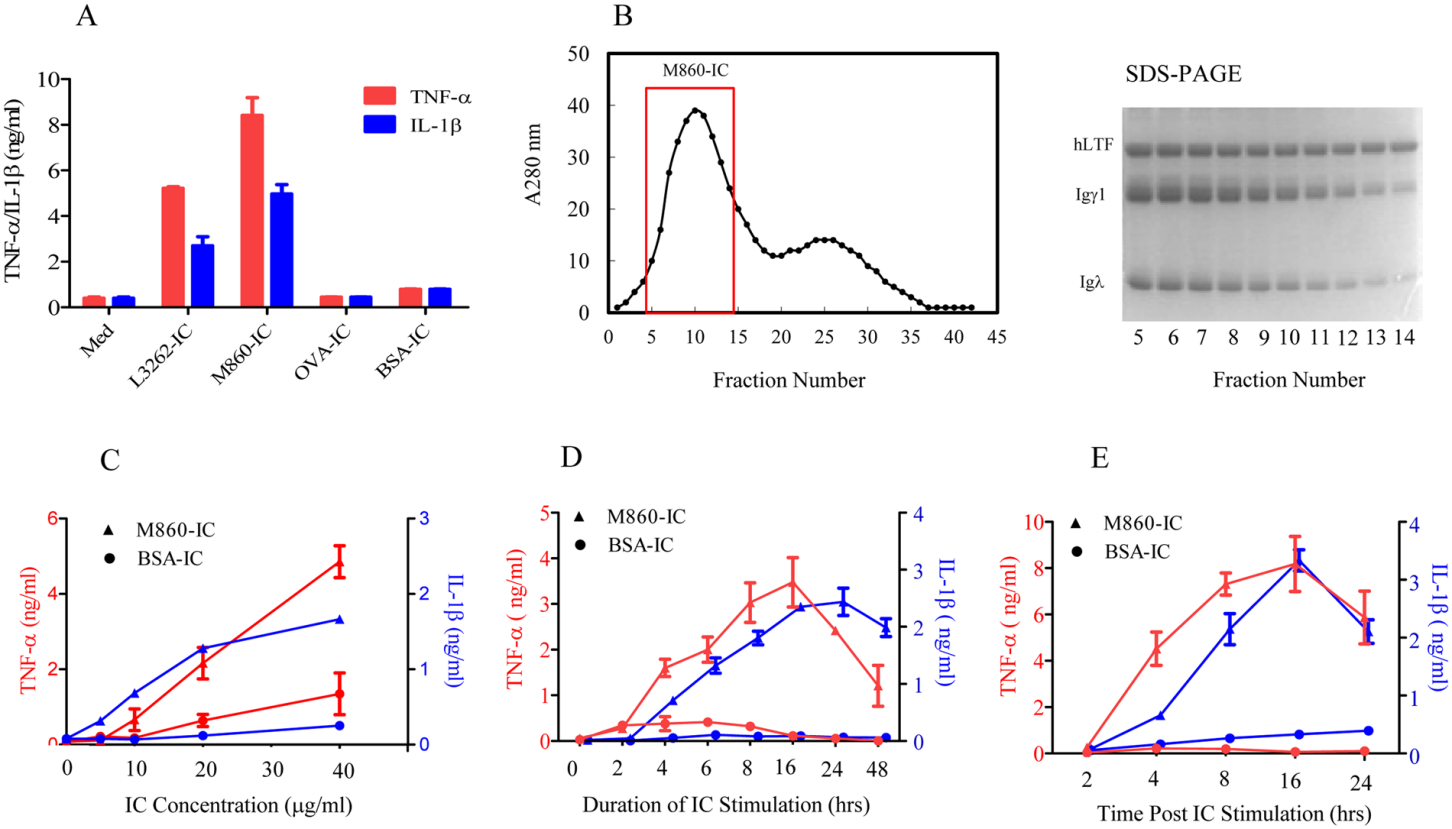
Functional comparison between LTF-ICs and control ICs. (**A**) Freshly fractionated human monocytes were stimulated with or without (*Med*) 15 μg/ml equal proportion mixtures of huLTF plus L3262 (*LTF-IC*), or huLTF plus mAb M860 (*M860-IC*), or OVA plus mAb M562 (*OVA-IC*), or BSA plus mAb J1 (*BSA-IC*) for 18 h. (**B**) For preparation of preformed M860-IC, 10 mg/ml of huLTF and M860 were mixed overnight and then fractionated using a Sephadex column, monitoring the fractions by spectrometry at 280 nm (histogram on the left). Guided by SDS-PAGE 10% gel electrophoresis results of the elution samples (image on the right), fractions 5–14 of the first peak were harvested and combined. (**C**) Human monocytes were stimulated with either increasing concentrations (5, 10, 20, 40 μg/ml) of preformed M860-IC, or preformed BSA-IC, for 18 h, or (**D**) with 30 μg/ml preformed M860-IC or BSA-IC for various length of time up to 48 h, or (**E**) with 30 μg/ml preformed M860-IC or BSA-IC for 1 h, washed and then cultured in fresh medium for various length of time up to 24 h. TNF-α (red lines or bars) and IL-1β (blue lines or bars) levels in culture supernatants were measured by ELISAs. Values are the mean concentration (ng/ml) ± SEM from triplicate cultures. Results are representatives of experiments performed at least three times.

Mouse mAbs against OVA (Clone M562) or bovine serum albumin (BSA) (Clone J1), produced in house and highly specific for the respective immunizing Ags and of IgG1 subclass, were employed for preparation of control ICs. While huLTF plus M860 (M860-IC) or huLTF plus L3262 (L3262-IC) elicited significant levels of TNF-α and IL-1β secretion by human monocytes *in vitro*, equal proportion mixtures (15 μg/ml for each protein) of OVA plus M562 (OVA-IC), or BSA plus J1 (BSA-IC) did not (Fig. 3A). Taking this further, preformed ICs of LTF-M860 and BSA-J1, fractionated using Sephadex columns and assayed using SDS-PAGE electrophoresis (Fig. 3B), were serially titrated against human monocytes for cytokine induction. The dose-response study again confirmed the potency and specificity of the LTF-IC in the induction of TNF-α and IL-1β secretion by human monocytes (Fig. 3C). Kinetic analysis showed that TNF-α and IL-1β secretion by human monocytes under M860-IC stimulation peaked at 16-18 h, whilst that in the BSA-IC group remained barely detectable throughout the 48 h period (Fig. 3D). Moreover, a 1 h pulse treatment with M860-IC, but not with BSA-IC, was sufficient to trigger TNF-α and IL-1β responses lasting over 24 h (Fig. 3E). Based on statistical analysis of results from several independent experiments, there is no significant difference between TNFα levels in monocyte cultures given 1 h pulse stimulation or in the presence of M860-IC for the entire duration.

### Synergistic effect of LTF-IC with LPS

**Figure 4:**
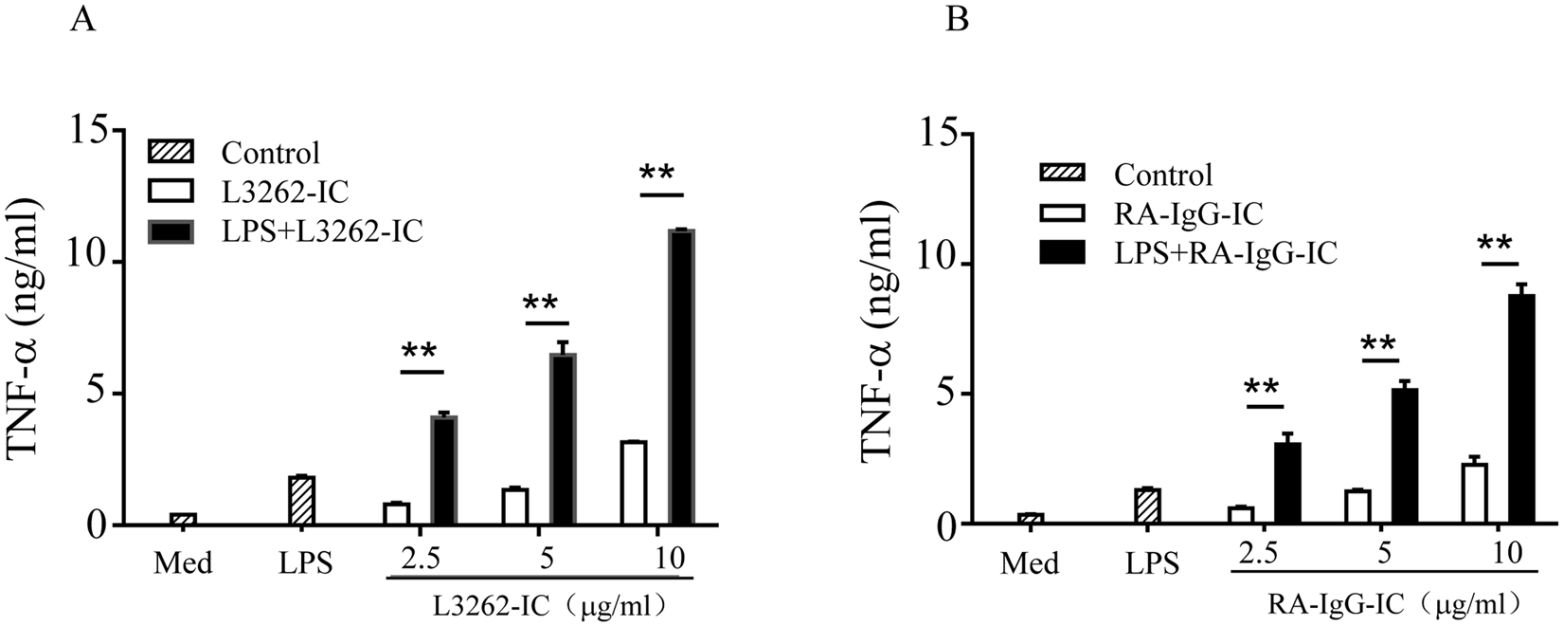
Synergistic effect of LTF-ICs with LPS. Freshly fractionated human peripheral blood monocytes were treated with various concentrations of either L3262-IC (**A**) or RA-IgG-IC (**B**), in the presence, or absence, of 1 μg/ml LPS (*LPS* + *L3262-IC*) for 18 h, followed by quantitation of TNF-α in the culture supernatant by ELISA. Cells maintained in medium only (*Med*) or stimulated with LPS alone (*LPS*) were included as controls. **p < 0.01 compared with cells treated with the L3262-IC only group. These are representatives of 3 independent experiments.

LTF is well known for its ability to sequester LPS activity though competing with soluble CD14 (sCD14) and/or LPS-binding protein (LBP) for LPS binding^3–6^ or counteracting the NF-κB signaling pathway associated with LPS activation of phagocytic cells^33–36^. Surprisingly, we found that LTF-ICs exhibited a clear synergistic effect with LPS in activating human monocytes. Human monocytes cultured with mixtures of suboptimal concentration LPS (1 μg/ml) and L3262-IC, or RA-IgG-IC, produced significantly more TNF-α than the additive effects of the two stimulants individually (Fig. 4A and B). As expected, neither huLTF nor LTF-Abs alone enhanced LPS activity in similar experiments (data not shown). These results indicate a synchronizing effect between signals triggered by LPS and LTF-ICs in monocytes.

### Requirement for CD32a (FcγRIIa), mCD14 and TLR4 in LTF-IC-induced human monocyte activation

**Figure 5:**
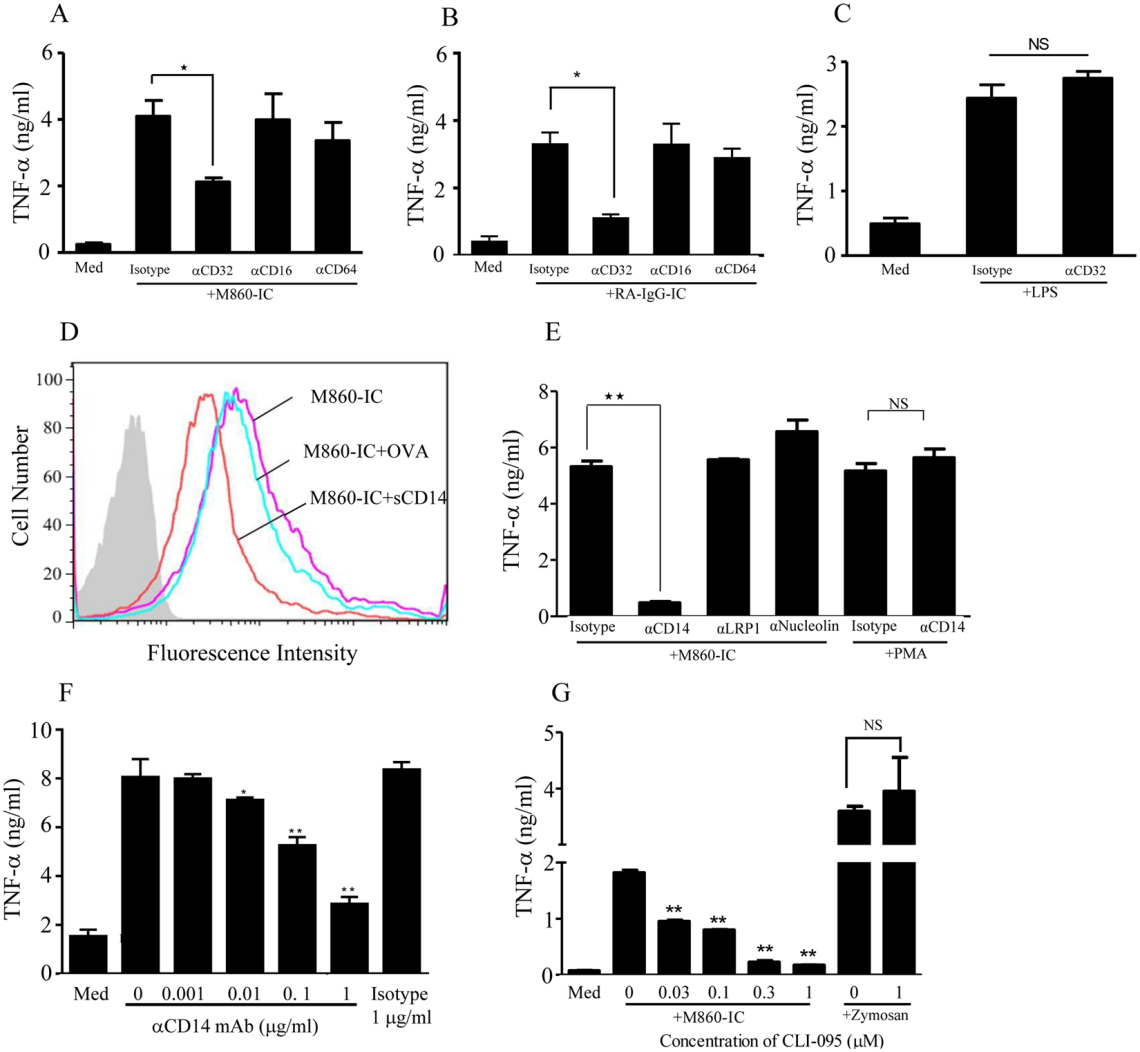
Role of CD32a and mCD14 in LTF-IC-mediated monocyte activation. Human monocytes were stimulated with 30 μg/ml huLTF-M860-IC (**A**), or RA-IgG-IC (**B**), in the presence or absence (*Med*) of blocking mAbs (15 μg/ml) against CD16 (B73.1, eBioscience), or CD32 (IV.3, eBioscience), or CD64 (10.1, Stem Cell Tech) for 18 h, followed by quantitation of TNF-α in the culture supernatant by ELISA. (**C**) Monocytes stimulated with 3 μg/ml LPS in the presence or absence (*Med*) of anti-CD32a mAb (15 μg/ml), or isotype control Abs as controls. (**D**) Human monocytes were stained with 30 μg/ml M860-IC containing FITC-conjugated huLTF in the presence or absence (*M860-IC*) of 10 μg/ml soluble recombinant human CD14 (*M860-IC* + *sCD14*) or OVA (*M860-IC* + *OVA*) for 1 h at 4 °C, followed by FACS analysis. Unstained cells were included as negative control (filled histogram). (**E**) Human monocytes were treated with 30 μg/ml huLTF-M860 IC in the presence of mouse mAbs (15 μg/ml) against human LRP-1 (α*LRP1*), nucleolin-1 (α*Nucleolin*), or CD14 (α*CD14*) for 18 h, followed by quantitation of TNF-α levels in the culture supernatants by ELISA. Cells treated with 1 μg/ml PMA in the presence of αCD14 mAb or isotype control mAb were included for specificity controls. (**F**) The anti-CD14 mAb was titrated against LTF-M860 IC (30 μg/ml) in monocyte activation assays with mouse IgG1 (1 μg/ml) as isotype control. Readings from unstimulated cells (*Med*) are also indicated in the figure. (**G**) TLR4-specific inhibitor CLI-095 was titrated against LTF-M860 IC (30 μg/ml) in monocyte activation assays monitoring TNF-α production. Monocytes stimulated with 5 μg/ml zymosan in the presence or absence of 1 μg/ml CLI-095 were included as specificity control. For ELISAs, values are mean TNF-α concentration (ng/ml) ± SEM from triplicate cultures. *p < 0.05, **p < 0.01 compared with M860-IC- or LPS-stimulated cells in the presence of isotype control Abs or in the absence of inhibiting agents. *NS*: not statistically significant. Results are representative of three independent experiments.

ICs are known to interact with the FcγR-expressing innate immune cells (e.g. DCs, monocytes/macrophages, neutrophils) via Fc portion of the IgH chain^22, 23^. Indeed, when mAb M860 was pretreated with papain or pepsin (both are capable of cleaving the Fc portion of IgG, Supplemental Figure S1A) its ability to synergize with huLTF in activating human monocytes was almost completely lost (Supplemental Figure S1B). To further identify the FcγR(s) required for LTF-IC function, blocking mAbs against FcγRI (CD64), FcγRIIa (CD32a), or FcγRIII (CD16) were used in functional inhibition assays. TNF-α production by monocytes stimulated with LTF-M860 ICs, or RA-IgG-IC, was significantly blocked by αCD32a, but not by αCD64 or αCD16, mAb (Fig. 5A and B). Note that, in previous studies, within the concentration range employed herein, the anti-CD16 and anti-CD64 mAbs exhibited strong suppression effect on monocyte activation triggered by CD16 and CD64 signaling, respectively^37, 38^. Additionally, CD32a blockade did not have any detectable effect on LPS-induced responses in similar experiment (Fig. 5C). These results suggest that CD32a, but not CD16 and CD64, is an indispensable surface receptor for LTF-IC-mediated monocyte/macrophage activation.

It has been shown that IC-mediated FcγR cross-linking alone is by far insufficient to trigger full activation of myeloid cells and result in their proinflammatory cytokine production^39, 40^. The potent monocyte/macrophage-stimulating activity of LTF-IC suggests that huLTF in the complex can also deliver activation signaling, presumably through direct interaction with cell surface LTF-Rs and/or intracellular sensors. Supported by an earlier report that huLTF can bind with a high affinity to sCD14 *in vitro* ^36^, we hypothesized that glycophosphotidyl inositol (GPI)-anchored CD14 (mCD14), a surface marker for phagocytic cells, is a likely receptor for LTF-ICs. Consistent with this notion, recombinant sCD14 effectively blocked the binding of FITC-labeled huLTF-ICs to human monocytes *in vitro* (Fig. 5D). Furthermore, mAb against human CD14, but not that against other potential LTF-Rs such as low-density lipoprotein receptor-related protein-1 (LRP-1) and nucleolin-1^41, 42^, almost completely abolished the monocyte-activating effect of LTF-ICs in functional assays in a dose-dependent manner (Fig. 5E and F). It should be emphasized that the inability of anti-LRP-1 and anti-nucleolin-1 Abs in preventing LTF-IC-mediated monocyte activation excludes the possibility that anti-CD14 mAb blocked LTF-IC function through its Fc fragment.

It has been shown that the GPI-anchored mCD14 relies on Toll-like receptor 4 (TLR4) for signaling following LPS ligation^43^. Interestingly CLI-095, a specific TLR4 inhibitor, was capable of suppressing LTF-IC-induced TNF-α production by human monocytes in a dose-dependent fashion, while it had no effect on the TNF-α production induced by zymosan which activates monocytes/macrophages through dectin-1 rather than TLR4^44^ (Fig. 5G). These results collectively suggest that LTF-ICs may employ, in addition to FcγRIIa, the CD14/TLR4 complex (major components of the LPS signaling machinery) as a key signaling receptor during monocytes/macrophages activation.

To exclude the possibility that mCD14 cross-linking alone by aggregated huLTF could activate monocytes/macrophages without FcγR participation, biotinylated huLTF (*LTF-Bio*), which could be cross-linked by avidin to form dimmers, tetramers and higher molecular weight oligomers, was used to treat human monocytes. As shown in Supplemental Figure S1C–E, when avidin was added to LTF-Bio-treated cells, no significant increase in intracellular TNF-α expression was observed, thereby supporting our “co-ligation” hypothesis that simultaneous engagement of FcγRIIa and mCD14 on the cell surface is key to the potent pro-inflammatory monocyte/macrophage-stimulating properties of LTF-ICs.

### Intracellular signaling mechanisms in monocytes following LTF-IC ligation

**Figure 6:**
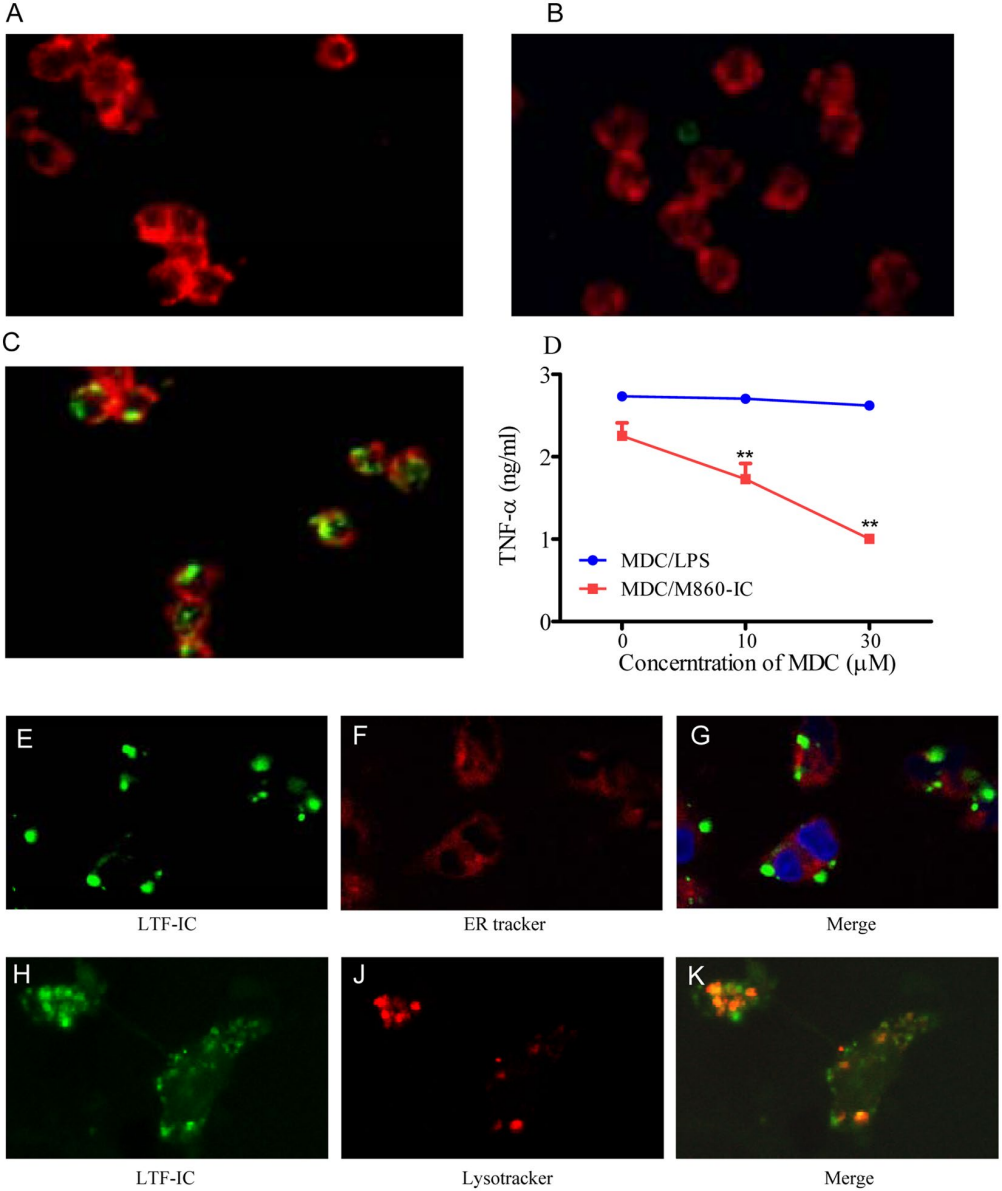
LTF-IC endocytosis by human peripheral blood monocytes. Human monocytes were sequentially stained with 15 μg/ml FITC-LTF (**A**), mixture of FITC-OVA plus mAb M562 (**B**), or FITC-LTF plus mAb M860 (**C**) for 1 h at 37 °C and for the second half h with PE-labeled mAb against human CD14. The cells were imaged with a Nikon confocal microscope system A1. In functional assays (**D**), the monocytes were stimulated with 30 μg/ml LTF-M860 IC (*MDC/M860-IC*) or 3 μg/ml LPS (*MDC/LPS*) in the presence or absence of MDC for 18 h, followed by quantitation of TNF-α in the culture supernatant by ELISA. Values are mean concentration (ng/ml) ± SEM from triplicate cultures. **p < 0.01 compared with the LPS/MDC group. For intracellular localization of the internalized LTF-ICs, human monocytes were stained with 30 μg/ml FITC-huLTF plus mAb M860 for 1 h at 37 °C, followed by treatment with red fluorescent ER tracker (**E**,**F**,**G**) or lysosome tracker (**H**,**J**,**K**). The specimens were observed under a confocal microscope through green and red channels, merged micrographs are shown on the right. Results are representative of 3 experiments.

**Figure 7:**
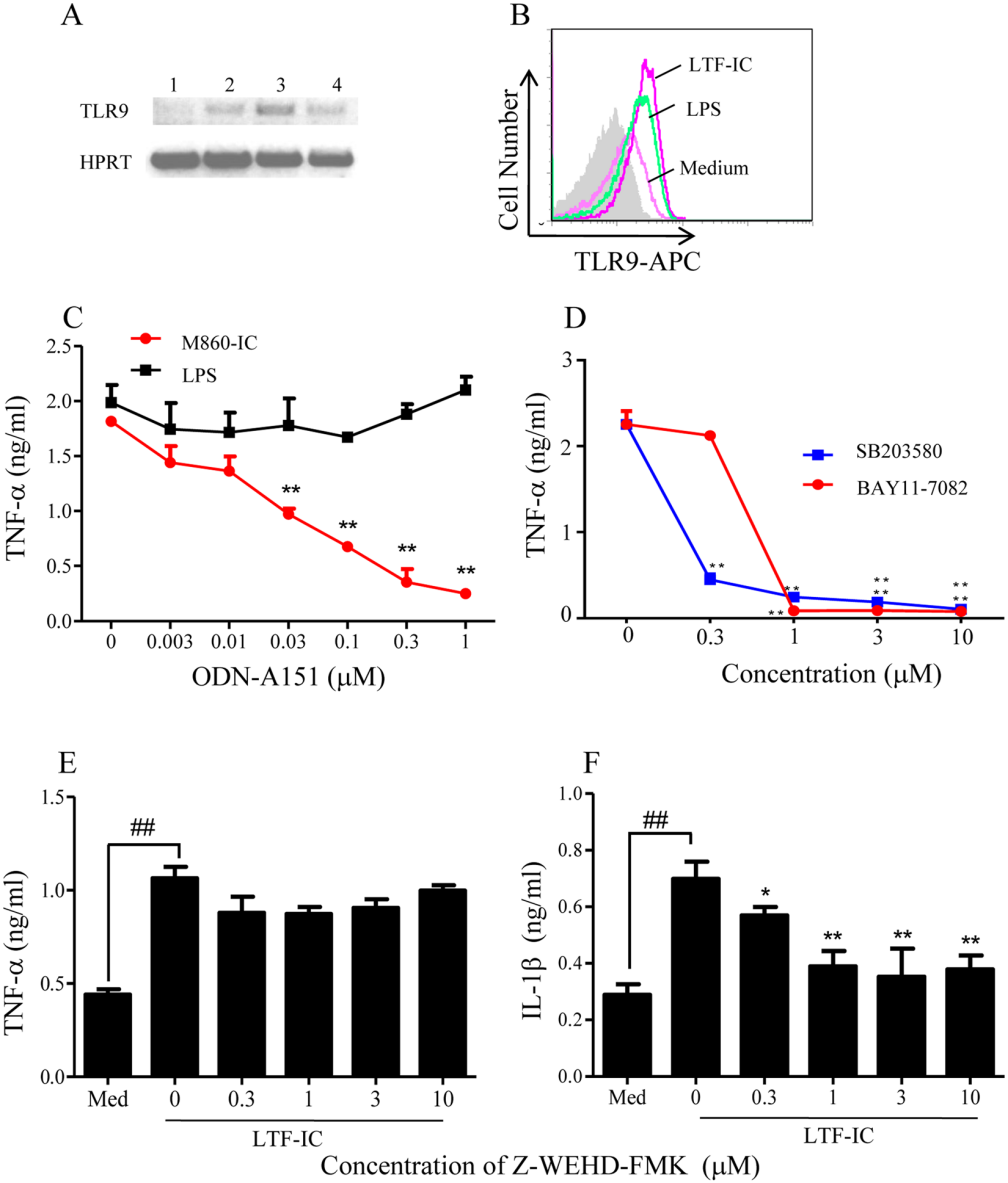
Signaling mechanisms triggered by LTF-IC in human monocytes. (**A**) RT-PCR of TLR9 or HPRT (control) mRNA from human monocytes stimulated with 30 μg/ml M860-IC for 0, 1, 3, and 18 h (lanes 1, 2, 3, and 4, respectively) *in vitro*. The full length gel was shown in Fig. S3. (**B**) Intracellular staining for TLR9 expression in human monocytes stimulated with 30 μg/ml LTF-IC or 3 μg/ml LPS for 18 h was carried out using APC-labeled mouse anti-human TLR9 mAb (eB72-1665). Unstimulated (*Medium*) and unstained cells (filled histogram) were included as controls. (**C**) TLR9-specific inhibitory ODN (ODN-A151) was titrated against human monocytes stimulated with 30 μg/ml M860-IC or 3 μg/ml LPS for 18 h and TNFα production determined by ELISA. Values are mean concentration (ng/ml) ± SEM from triplicate cultures. **p < 0.01 compared with the LPS-stimulation without inhibitor. (**D**) Chemical inhibitors specific for NF-κB (BAY11-7082) or p38 MAPK (SB203580), respectively, were titrated against human monocytes stimulated with 15 μg/ml M860-IC for 18 h and TNF-α production determined by ELISA. (**E**,**F**) Human monocytes were stimulated with 15 μg/ml M860-IC in the presence or absence of increasing concentration of caspase-1 inhibitor (Z-VEHD-FMK) for 18 h, followed by quantitation of TNF-α (**E**) and IL-1β (**F**) in the culture supernatant by ELISA. Unstimulated cells cultured in medium only (*Med*) were included as controls. *p < 0.05, **p < 0.01 compared with LTF-IC stimulation without inhibitor. ^##^ *p* < 0.01 compared with unstimulated cell control. Results are representative of 3 experiments.

The kinetic study results (Fig. 3E) indicate that, following colligation with LTF-Rs and FcγRs on the surface of the responding cells, LTF-ICs could be internalized and continue to function through intracellular sensors. Indeed, FITC-huLTF-containing ICs were efficiently internalized by human monocytes following 1 h incubation at 37 °C, whilst FITC-huLTF alone, or FITC-OVA-IC, was poorly captured and internalized in the same experiment (Fig. 6A–C). Moreover, MDC, a potent chemical inhibitor of endocytosis, suppressed TNF-α production by human monocytes *in vitro* (Fig. 6D). It is also of interest to note that the internalized LTF-IC (or LTF thereof) co-localized with lysosomes, but not ER, of the cell (Fig. 6E–K). Means *et al*. showed that DNA-containing SLE-ICs transiently co-localized to intracellular lysosomes containing FcγRIIa and TLR9, thereby defining a pathway in which FcγRIIa delivers SLE-ICs to intracellular compartment containing TLR9 in DCs^30^. We reasoned that a similar pathway could be employed in LTF-IC-mediated monocyte/macrophage activation. Although TLR9 expression in resting monocytes is low, but it is significantly increased following LTF-IC activation as revealed by semi-quantitative RT-PCR analysis on TLR9-encoding mRNA (Fig. 7A) and also intracellular staining for TLR9 protein and FACS analysis (Fig. 7B). Importantly, ODN-A151, a suppressive ODN capable of competitively inhibiting TLR9, effectively down-regulated TNF-α production by human monocytes cultured with M860-IC (Fig. 7C). Furthermore, TNF-α production by LTF-IC-activated monocytes was also blocked by inhibitors against p38 MAPK (SB203580) and NF-κB (BAY11-7082) dose-dependently (Fig. 7D), providing additional evidence for the involvement of LPS signaling pathways in LTF-IC-induced monocyte activation. It has recently been reported that IL-1β secretion by monocytes/macrophages depends on inflammasome activation^45^, of which caspase is a key enzyme responsible for pro-IL-1β cleavage. Interestingly, Z-WEHD-FMK, a specific caspase-1 inhibitor, strongly suppressed IL-1β, but not TNF-α, production by human monocytes under stimulation with LTF-ICs (Fig. 7E and F).

## Discussion

In this report we have shown a positive correlation between the prevalence of circulating anti-LTF IgG autoantibodies and RA in humans (Fig. 1), albeit there is much variation in the levels of anti-LTF autoantibodies in the RA sera and a small percentage of healthy subjects also have relatively high titer serum IgG against LTF. Some autoantibodies such as rheumatoid factors (RF) and anti-citrilinated protein Abs are valuable for clinical diagnosis of RA. However, whether LTF-Abs are also valuable as such is currently unclear. It would be of great interest to analyze if there is clear correlation between clinical indicators (e.g. disease severity, drug usage) and LTF-specific autoantibody levels, much larger sample sizes of RA sera would be required to address these questions in full. We are also developing assay systems for simple and rapid quantitation of LTF-ICs in human sera, which may become useful for further studies in this direction.

The main finding of the present study is that ICs between huLTF and LTF-specific IgG from RA patients are potent activators of human monocytes/macrophages via co-ligation of CD14 and FcγRIIa in an inernanlization-, TLR4- and TLR9-dependent manner. Given that LTF concentration can be elevated significantly in damaged tissues^20, 21^, the concentration range of LTF-ICs (10–30 μg/ml) used in this study is pathophysiologically relevant. LTF-ICs containing IgG from mice and rabbits are as effective as that containing human Abs in activating human monocytes/macrophages *in vitro*. This can be explained by the fact that rabbit IgG and mouse IgG1 in ICs can bind human FcγRs with relatively high affinity^22, 23^. In order to further elucidate the roles of LTF-ICs in autoimmune arthritis, *in vivo* experiments employing relevant animal models (e.g. collagen-induced arthritis in mice) would be needed. Given that FcγRIIa exists in humans but not in mice, such experiments would require hFcγRIIA-trangenic animals. Interestingly, hFcγRIIA-trangenic mice, prepared by Tan Sardjono *et al*., develop spontaneous multisystem autoimmune disease and hypersensitivity to antibody-induced inflammation^46^.

The proinflammatory properties of LTF-ICs are unique amongst endogenous ICs as activators of innate immune cells in the following aspects: (i) LTF-ICs are over 10 folds more potent than ordinary ICs in eliciting proinflammatory cytokine responses in human monocytes/macrophages; (ii) A brief pulse (30 min) with LTF-ICs causes long lasting cytokine responses in human monocytes; (iii) In contrast to free huLTF which is capable of sequestering LPS activity *in vitro*, LTF-ICs synergize with LPS in activating human monocytes/macrophages. Our results, together with recent advances endorsing pivotal roles for ICs in the development of RA^28–31^, may help us to better understand the pathogenesis of autoimmune arthritis. Since LTF-Abs are found in patients with various systemic autoimmune diseases^13–19^, a similarly important role of LTF-ICs in a much wider spectrum of inflammatory autoimmune disorders can be anticipated.

**Figure 8:**
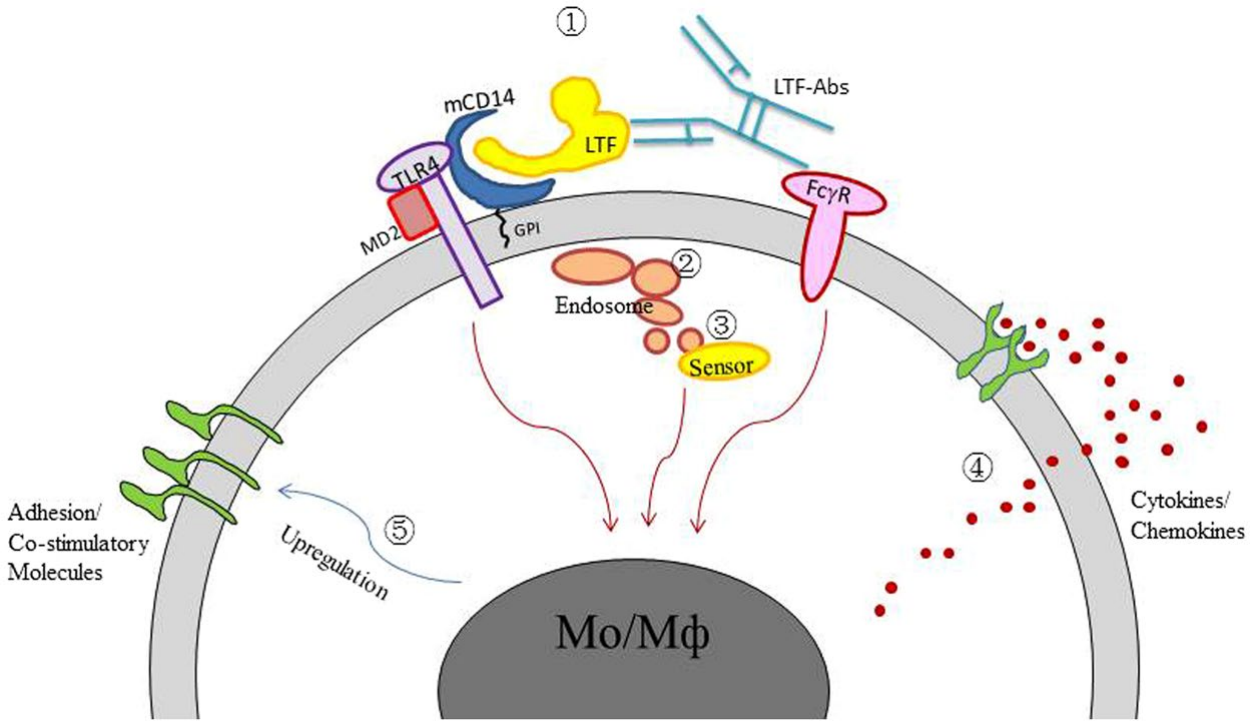
Schematic representation of the dual-ligation model. LTF-ICs may activate monocytes/macrophages via the following steps/pathways: ① Simultaneous ligation of mCD14/TLR4 and FcγRIIa; ② Endocytosis of the captured LTF-ICs; ③ The endocytosed LTF-ICs gaining access to lysosomes and contact with intracellular sensors (e.g. TLR9) and/or inflammasome, which will result in further signaling cascade activation; ④ Pro-inflammatory cytokine secretion; ⑤ Potential up-regulation of adhesion molecules and pattern recognition receptors. The activation signals from CD32a, mCD14/TLR4 and intracellular sensors are expressed by red arrows. Likely interaction and cross-talk between them underlines the potent pro-inflammatory properties of LTF-ICs.

Membrane-bound CD14 is apparently a major surface receptor for LTF-ICs. We found that the monocyte-binding and -activating effects of LTF-ICs are susceptible to heparin inhibition (Supplemental Figure S2), which is in agreement with previous reports that LTF bound to monocytic cells as well as various cationic ligands such as DNA and LPS in a heparin-sensitive way^6, 36, 47^. The GPI-anchored CD14 molecule constitutively resides in lipid rafts (microdomains) on the membrane surface of monocytes/macrophages^43^. Following LPS ligation, adaptor and signaling molecules such as TLR4 and MD2 are recruited to the site of LPS ligation within the lipid rafts, resulting in the formation of LPS sensing and signaling complex (LSSC) responsible for an enhanced (focused) signaling event though the NF-kB and MAPK pathways^43^. Since CD14 and TLR4 are the major components of LSSC in lipid rafts, we propose that the LPS signaling mechanisms are triggered during LTF-IC activation of monocytes/macrophages and also that LTF-IC-mediated cross-talk between CD32a and LSSC (or related receptor complex) acts as a strong signal for monocytic cell activation and subsequent internalization and intracellular triggering processes. A schematic representation of the co-ligation model is presented in Fig. 8. Following internalization, huLTF (or fragments thereof) gains access to intracellular sensors such as TLR9 and inflammasome, resulting in the enhanced functional state of the cell. Thus, LTF-IC-activated monocytes and macrophages in the joints could play significant roles in the pathogenesis of RA by secreting large amounts of pro-inflammatory cytokines such as TNF-α and IL-1β. High concentration of LTF found in synovial fluid of RA patients^20^ is a likely result of neutrophil accumulation and activation because LTF is a major component of secondary granules of neutrophils^1^. Based upon these observations, we further hypothesize that factors in RA synovium including LTF-IC, monocyte/macrophage activation, proinflammatory cytokine secretion, neutrophil accumulation/triggering, LTF release and additional LTF-IC formation will form a positive-feedback loop leading to arthritogenic damage *in vivo*.

An interesting analogy can be drawn between LTF-ICs and DNA-containing ICs (DNA-ICs). In the case of DNA-IC-mediated DC activation, the internalized CpG-containing dsDNA triggers activation cascade through cooperation of CD32 and TLR9^28, 30^. Another case in point is RA-specific autoantibodies to citrullinated proteins complexed with fibrinogen, which could induce macrophage secretion of TNF-α through FcγRIIa and TLR4 engagement^28, 29, 31^. It will be of considerable interest to examine if such immunologically active ICs could collaborate (synergize) with each other to endorse further enhanced inflammatory reactions, as seen in the case of LTF-IC-pretreated monocytes responding more vigorously to stimulation with otherwise non-stimulatory OVA-IC (data not shown).

The inhibitory hFcγRIIb is expressed only poorly on 20% blood monocytes^23, 48^, therefore its potential influence on *in vitro* studies employing blood monocytes and monocyte-derived macrophages is negligible. However, it is also evident that tissue macrophages may express hFcγRIIb at higher level^49^, therefore more complex intracellular signaling through FcγRIIa as well as FcγRIIb could be triggered by LTF-IC in synovial macrophages. To further elucidate the role of LTF-IC in autoimmune arthritis, *in vivo* experiments employing hFcγRIIA-trangenic and/or FcγRIIb-knockout mice would be needed.

Taken together, LTF-ICs can be considered novel proinflammatory mediators that can elicit strong proinflammatory cytokine production by monocytes/macrophages, thereby contributing to the pathogenesis of the autoimmune diseases such as RA. This idea should facilitate the development of therapies of autoimmune diseases with specific targets including the binding sites, the dual ligation requirement, the internalization steps, and the intracellular sensors in LTF-IC-mediated activation of innate immune cells.

## Materials and Methods

### Antibodies and reagents

Monoclonal Ab M860 was prepared in our laboratory as described elsewhere^32^. Monoclonal Abs M562 and J1 against OVA and BSA (Sigma), respectively, were also generated in this laboratory using the same procedure. For preparation of anti-huLTF IgG from RA patients (RA-IgG), IgG from 6 pooled plasma samples shown by ELISA to contain high levels of anti-LTF antibodies were sequentially purified by affinity chromatography on LTF-S4B (prepared in our laboratory) and Protein A-S4B columns (Pierce). The eluted IgG fractions were concentrated by centrifugation with buffer exchange to PBS (Amicon Ultra, Millipore) and were depleted of endotoxin by filtration through a polymyxin B column (Detoxigel). IgG concentrations were determined by optical density at 280 nm; IgG was aliquoted, and stored at −80 °C. For preparation of preformed LTF-ICs, huLTF (2 mg/ml) and M860 (2 mg/ml) were mixed in a sterile tube with gentle rotation at 37 °C for an hour. The mixture was then loaded onto a Sephadex Superfine G-75 column for separation of the IC from the uncoupled Ab and antigen. IC was eluted by 0.9% NaCl solution and the elution was collected every 0.5 ml. Samples of the elutions were separated by SDS-PAGE and stained with Coomasie blue. The elutions of IC were pooled, desalted and concentrated. Endotoxin was removed by polymyxin B coupled beads repeatly and the level of endotoxin in IC is below 1 EU/mg which was detected by Chromogenic LAL Endotoxin Assay Kit (Genscript). Control ICs between BSA and J1 were prepared similarly.

Rabbit anti-huLTF polyclonal IgG (L3262), rabbit IgG, huLTF, LPS, OVA, heparin, FITC, MDC and zymosan were from Sigma-Aldrich. PE-labeled anti-CD11b and Alexa Fluor 647-labeled anti-human CD14 mAbs were from Biolegend. APC-labeled mouse anti-human TLR9 mAb (eB72-1665) was from BD Biosciences. Mouse mAbs against human CD16 (B73.1) or CD64 (10.1) were from eBioscience. Mouse anti-human mAb CD32a (IV.3) was from Stem Cell Technologies. Mouse anti-human CD14 mAb (134620) and Z-WEHD-FMK were from R&D. Mouse mAbs against human nucleolin-1 and LRP1 were from Santa Cruz. F(ab)’_2_ and Fab were prepared using immobilized pepsin and papain (Thermo scientific). Recombinant soluble human CD14 was from Peprotech. Biotin and avidin were from Thermo Scientific. ODN-A151 and CLI-095 were from Invivogen, BAY11-7082, SB203580, lysotracker and ER tracker were from Beyotime.

### Sample collection

Peripheral blood was collected from patients with RA (n = 80; 20–93 y of age; mean = 57.4 y of age; 48 females) attending the Department of Rheumatology, Peking University people Hospital (Beijing, China) between 2008 and 2009. Patients were diagnosed according to the 1987 criteria of the American College of Rheumatology^50^. All blood samples were collected while their disease was in an active phase. The blood samples were processed within 18 h of collection and the cell-free sera were stored at −80 °C until use. Serum samples from healthy volunteers between 20 and 50 y of age (n = 35, mean age, 30.6 y) were included as controls.

### Cell isolation and culture

For preparation of monocytes from human peripheral blood mononuclear cells (PBMCs), venous blood from healthy donors were 2-fold diluted using RPMI 1640 medium and overlaid on Ficoll lymphocyte separating solution (Sigma-Aldrich) followed by centrifugation at 400 × g for 30 min at room temperature. Cells in the interphase were collected and washed. Monocytes were isolated using Monocyte Isolation Kit (Miltenyi Biotec) according to manufacturer’s instructions of positive and negative selection. The resultant cells were 90% CD14-positive and viable as evidenced by flow cytometric analysis and microscopic observation. Purified monocytes were cultured in “R10” medium: RPMI 1640 supplemented with 10% (v/v) Fetal calf serum (FCS, HyClone Laboratories), penicillin/streptomycin (100 U/ml), L-glutamine (2 mM), and 2-ME (5 × 10^−5^ M). Cells were cultured at a concentration of 5 × 10^5^ cells/ml of medium at 37 °C and 5% CO_2_. For generation of monocyte-derived macrophages, human monocytes were differentiated into macrophages by culture for 7 days in RPMI containing 10% FCS and 50 ng/ml human M-CSF.

### Monocyte/macrophage stimulation

Freshly isolated human monocytes (1 × 10^5^), or monocyte-derived macrophages, were incubated with huLTF, LTF-Abs (of different sources), mixture of huLTF plus LTF-Abs, preformed huLTF-ICs, chicken ovalbumin (OVA)-ICs or BSA-ICs for 16–18 h, after which TNF-α levels in culture supernatants were determined by ELISA. The TLR-4 ligand LPS (3 μg/ml) was used as control for TNF-α induction. ICs were also generated *in vitro* by incubation of huLTF with specific IgG Abs or, as a control, with normal polyclonal rabbit IgG (Dako Cytomation) or irrelevant mIgG1 (prepared in this laboratory) at 37 °C for 45 min.

### ELISAs

The concentration of human TNF-α and IL-1β in the culture supernatant was determined using ELISA kits (BioLegend) following the manufacturer’s instructions. Standard curves were established using human recombinant cytokines, and the assay detection limit was 7.8 pg/ml for TNF-α, 2 pg/ml for IL-1β. For detection of anti-huLTF autoantibodies in sera, ELISA plates were pre-coated with huLTF (2 µg/ml) in carbonate buffer (pH 9.6) at 4 °C overnight followed by incubation with blocking solution (1% BSA in PBS) for 2 h at 37 °C. The wells were washed five times with PBS containing 0.05% Tween 20 (PBST) and then 100 µl of diluted human sera were added in triplicates and incubated for 2 h at 37 °C. After five washes with PBST, the plates were incubated with HRP-labeled goat anti-human IgM or IgG Abs (Southern Biotech) for 1 h at 37 °C. The reaction was developed with 100 µl of O-phenylenediamine (OPD) (Sigma-Aldrich) for 5 min and stopped with 100 µl of 2 M H_2_SO_4_. OD was measured at 492 nm in an ELISA spectrophotometer (Titertek Multiscan Plus MK II; ICN Flow Laboratories).

### Fluorescence staining and FACS analysis

For indirect staining, human monocytes (1 × 10^6^ cells/tube) were incubated for 30 min at 4 °C with 20 µl of FITC-conjugated mAb against human CD14 (BioLegend) or FITC-conjugated isotype control mouse Abs (BioLegend). After washes, the cell pellets were re-suspended in 50 µl L3262-IC (equal proportion mixture of FITC-huLTF plus L3262), or M860-IC (equal proportion mixture of FITC-huLTF plus M860), or similar mixture of FITC-LTF plus isotype control Abs for 1 h at 4 °C.The cells were then washed three times in PBS and re-suspended in 1 ml staining buffer (PBS supplemented with 0.1% NaN_3_ and 0.2% BSA) for flow cytometric analysis on a FACS Calibur (Becton-Dickinson) gating on 5000 live cells. Data were analyzed by CellQuest software (Becton Dickinson).

### Confocal laser scanning microscopy

Monocytes (1.5 × 10^5^/well) were plated onto poly-l-lysine coated glass slides and allowed to adhere. The cells were then incubated in a total volume of 200 μl 0.5% BSA in PBS with 15 μg/ml FITC-LTF, or mixture of FITC-LTF plus mAb M860, or FITC-OVA plus mAb M562 for 1 h at 37 °C. After washing to remove unbound proteins, the cells were further stained with either PE-labeled anti-CD14 Abs or fluorescent trackers for lysosome or endoplasmic reticulum (ER), followed by 1% paraformaldehyde fixation and 1 μg/ml DAPI counterstain. The cells were imaged with a Nikon confocal microscope system A1.

### Statistical analysis

Difference between the values of the mLTF-IC and those of control groups were compared with the repeated measures ANOVA and Bonferroni’s multiple comparison post-hoc test. For experiments containing two groups, the differences were analyzed using the independent samples t test or two-sided, paired t test. Differences were considered statistically significant at p < 0.05. Statistical analysis was performed using SPSS 14.0 program (SPSS, Chicago, IL).

### Study approval

This study was approved by the Ethics Committees of Peking University Health Science Center, Peking University, Beijing and Soochow University Medical School, Suzhou, China. The methods were carried out in accordance with the guidelines of Peking University and Soochow University. Written informed consent was obtained from all participants prior to inclusion in the study.

## Electronic supplementary material

Supplemantary Materials
