# Author Correction: Extraordinarily potent proinflammatory properties of lactoferrin-containing immunocomplexes against human monocytes and macrophages

**DOI:** 10.1038/s41598-018-35598-8

**Published:** 2018-11-16

**Authors:** Lulu Hu, Xiaomin Hu, Kai Long, Chenhui Gao, Hong-Liang Dong, Qiao Zhong, Xiao-Ming Gao, Fang-Yuan Gong

**Affiliations:** 1 Institute of Biology and Medical Sciences, School of Biology and Basic Medical Sciences, Soochow University, Suzhou, China; 2 Department of Immunology, Peking University Health Science Center, Beijing, China; 3 Department of Physiology, Jiujiang College, Jiangxi Province, China

Correction to: *Scientific Reports* 10.1038/s41598-017-04275-7, published online 26 June 2017

The Acknowledgements section in this Article contains errors.

“This study was supported by grants from the Key Research Programs of Ministry of Science and Technology of China, National Foundation of Natural Science of China (31070781), Priority Academic Program Development of Jiangsu Higher Education Institution (PAPD), and Program for Changjiang Scholars and Innovative Research Team in University, Ministry of Education, China (IRT1075).”

should read:

“This study was supported by grants from National Foundation of Natural Science of China (31570868), Priority Academic Program Development of Jiangsu Higher Education Institution (PAPD), and Program for Changjiang Scholars and Innovative Research Team in University, Ministry of Education, China (IRT1075).”

