# Supplementary material for: Extraordinarily potent proinflammatory properties of lactoferrin-containing immunocomplexes against human monocytes and macrophages
Source: Sci Rep. 2017 Jun 26;7:4230. doi: 10.1038/s41598-017-04275-7 (PMC5484712; doi:10.1038/s41598-017-04275-7)
Supplement: Supplementary file 1 — Supplemantary Materials [file 41598_2017_4275_MOESM1_ESM.doc]

**Extraordinarily potent proinflammatory properties of lactoferrin- containing immunocomplexes against human monocytes and macrophages**

**Lulu Hu ^1^,** **Xiaomin Hu^1^, Kai Long^2, 3^, Chenhui Gao^1^, Hong-Liang Dong^1^, Qiao Zhong^1^, Xiao-Ming Gao^1^ & Fang-Yuan Gong^1^**

^1^Institute of Biology and Medical Sciences, School of Biology and Basic Medical Sciences, Soochow University, Suzhou, China

^2^Department of Immunology, Peking University Health Science Center, Beijing, China

^3^Department of Physiology, Jiujiang College, Jiangxi Province, China

**Correspondence**: Fang-Yuan Gong or Xiao-Ming Gao, School of Biology and Basic Medical Sciences, Soochow University, 199 Ren’ai Road, Suzhou 215123, China.

Tel. (86) 512 65882613. Email: xmgao@suda.edu.cn; [gongfangyuan@suda.edu.cn](mailto:gongfangyuan@suda.edu.cn)

**Supplementary Figures**


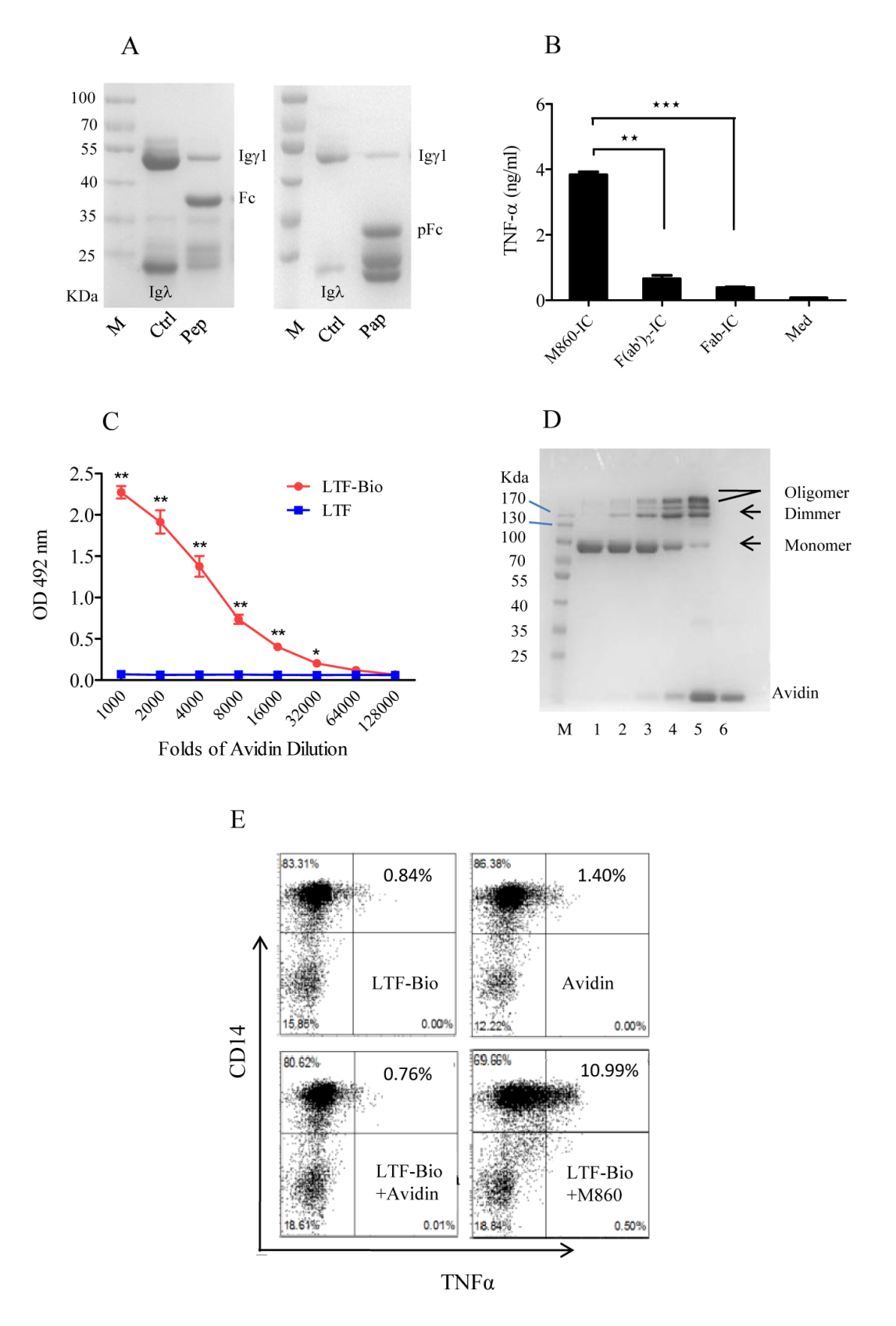


**Supplemental Figure S1.** **Dependence on IgG Fc in LTF-IC-mediated monocyte activation.** (**A**) mAb M860 (1 mg/ml) was digested with 10 units/ml pepsin (*Pep*) or papain (*Pap*) for 2 h at 37^0^C followed by Coomassie blue-stained SDS-PAGE 10% gel electrophoresis, with undigested mouse IgG1 included as controls (*Crtl*). The resultant Fc fragments and remaining residual Igγ1 chains in the digestion mixture are indicated. (**B**) Freshly prepared human monocytes were stimulated with or without (*Med*) equal mixtures of huLTF and M860 (*M860-IC*), huLTF and pepsin-digested M860 (*F(ab’)_2_-IC*), or huLTF and papain-digested M860 (*Fab-IC*), for 18 h followed by ELISA quantitation of TNF-α in the culture supernatant. Total concentration of the stimulatory proteins was 30 μg/ml in all groups. (**C**) Confirmation of successful biotinylation of huLTF. HRP-labeled avidin (0.1 mg/ml) was doubling diluted and dispensed in triplicate wells of ELISA plate pre-coated with biotinylated huLTF (*LTF-Bio*) or huLTF (*LTF*), with OPD as substrate. OD was measured at 492 nm in an ELISA spectrophotometer, values are the mean ± SEM from triplicate cultures. **p*<0.05, ***p*<0.01 compared with LTF stimulation group. (**D**) Coomassie blue-stained native PAGE 10% gel showing that, in the presence of increasing concentration of avidin (0, 10, 20, 40, 80, 160 ng/ml in lanes 1-6, respectively), LTF-Bio formed dimmers, tetramers as well as higher molecular weight oligomers. (**E**) Human monocytes were incubated with 10 μg/ml LTF-Bio for 1 h, followed by 30 ng/ml avidin (*LTF-Bio+Avdin*) or 30 μg/ml M860 (*LTF-Bio+M860*) for 18 h. Cells treated with LTF-Bio or avidin (upper left and upper right, respectively) only were included as negative controls. TNF-α expression by the stimulated cells was determined by intracellular staining with FITC-labeled anti-TNF-α mAb and also PE-labeled mAb against CD14 and FACS analysis. Percentages of CD14 and intracellular TNF-α double positive cells are indicated in the dot plots. Results are representative of experiments performed at least twice.

**
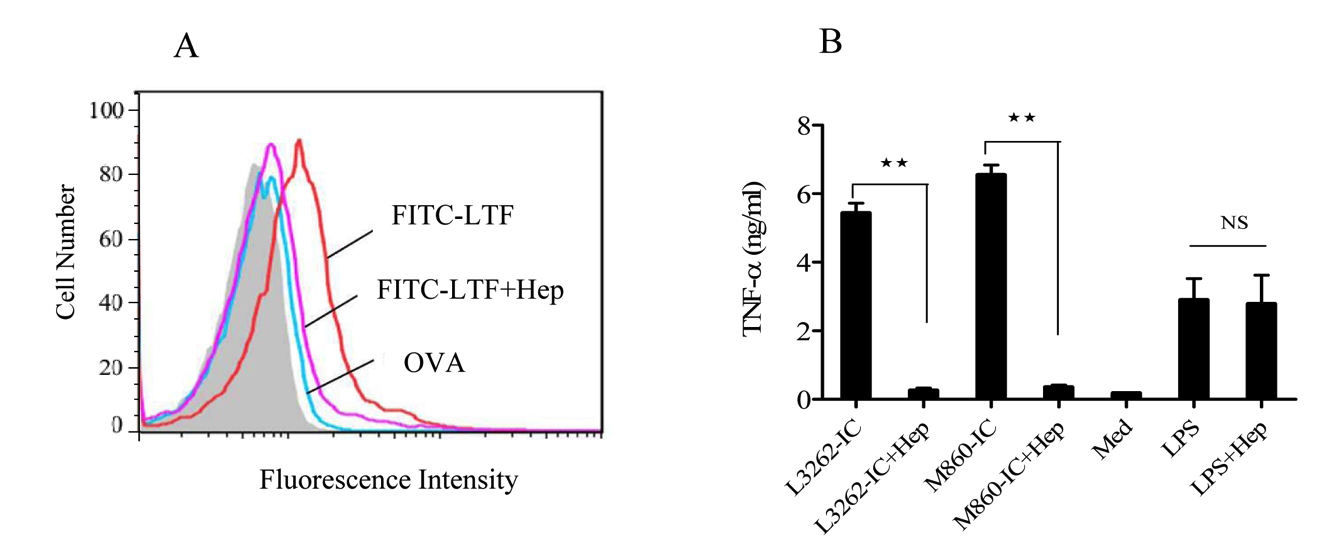
**

**Supplemental Figure S2. Heparin blocks LTF-monocyte binding and inhibits LTF-IC-induced monocyte-activation.** (**A**) Freshly prepared human monocytes were stained with FITC-labeled huLTF in the presence or absence (*LTF*) of 10 μg/ml heparin (*LTF+Hep*) for 1 h at 4^0^C followed by FACS analysis. FITC-labeled OVA was also included as specificity control. (**B**) Purified human monocytes were stimulated with 30 μg/ml L3262-IC, or M860-IC in the presence or absence of 10 μg/ml heparin for 18 h. Cells in culture medium alone (*Med*) or stimulated with 3 μg/ml LPS in the presence or absence of heparin were included as controls. TNF-α levels in culture supernatants were quantitated by ELISA. Values are mean concentration (ng/ml) ± SEM from triplicate cultures. ***p*<0.01, NS: not significant.


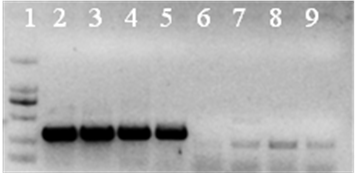


**Supplemental Figure S3** The invert picture of full-length gels shown in Fig. 7A. Lane 1, DL2000; Lane 2-5, HPRT；Lane 6-9，TLR9.
